# Optimal torque distribution of distributed driving AGV under the condition of centroid change

**DOI:** 10.1038/s41598-021-01038-3

**Published:** 2021-11-01

**Authors:** Wei Liu, Ping Liu, Yue Yu, Qingjie Zhang, Yidong Wan, Miufang Wang

**Affiliations:** 1 School of Automotive Engineering, Yancheng Institute of Technology, Yancheng, 224051 China; 2 Jiangsu Coastal Institute of New Energy Vehicle, Yancheng, 224051 China

**Keywords:** Engineering, Electrical and electronic engineering, Mechanical engineering

## Abstract

The torque distribution is researched under the condition of the centroid position of distributed drive automatic guided vehicle (AGV) with load platform and is uncertain due to the unknown movable load. The whole vehicle model under centroid variation, the efficiency model of the hub motor and the torque distribution control strategy based on a PID neural network are established. A hierarchical controller is designed to accurately ensure the economy and stability of the vehicle. Simulations of the proposed control strategy are conducted, the results show that the total power and lateral deviation distance of the driving wheels are reduced by 17.63% and 61.54% under low load conditions and 15.54% and 61.39% under high load conditions, respectively, compared with those of the driving wheels under the average torque distribution, and the goal of close slip rates of the driving wheels is achieved. A system prototype is developed and tested, and the experimental results agree with the simulation within error permissibility. The margin of error is less than 5.8%, the results demonstrate that the proposed control strategy is effective. This research can provide a theoretical and experimental basis for the torque optimization distribution of distributed drive AGVs under centroid variation conditions.

## Introduction

Distributed drives represent a new form of electric vehicle (EV) dynamics control^1^. These drives include a shorter driving chain and higher transmission efficiency, can easily independently control each wheel, and distribute the torque on each wheel in any proportion. These drives have important implications for improving the safety, stability, energy savings and other motion performance factors of vehicles, so they are ideal motion platforms for intelligent vehicles. Distributed drive EVs are becoming a popular topic in academic fields because of increasingly severe environmental and energy issues^2,3^.

Distributed drive AGVs constitute most of the vehicles in intelligent plants, improving transportation efficiency, and the research object of this paper is a distributed drive AGV with a variable centroid. The unknown movable load means the centroid of the AGV is uncertain, so the average torque distribution control strategy suitable for general vehicles is no longer applicable. Under this complex and uncertain condition, improving the motion control performance of the AGV is of great significance. The driving torque control strategy is directly related to the vehicle performance. An active control strategy is used for torque distribution to ensure the stability and economy of distributed drive vehicles and to give full play to the advantages of four-wheel drive torque control alone. Many scholars have done much research in this field.

A newly developed offline optimization method was used to evaluate the different objective functions for the torque distribution of four-wheel drive vehicles^4^. The experimental results indicated that the objective function based on minimum tire slip has better control performance than the method based on energy efficiency. Lenzo studied the influence of torque distribution on handling stability and showed that the front wheel drive force makes a greater contribution to the yaw moment^5^. A torque distribution control strategy based on wavelet control was proposed for EVs^6^. The proposed strategy not only enhances the stability on split roads but also supplies a smoother and faster torque response. A torque vector control system was designed to improve vehicle stability^7^. Safety-oriented layer control strategies have been proposed to improve the stability of distributed drive EVs^8,9^. A torque distribution method for electric off-road vehicles based on the load ratio of the front wheels to the rear wheels was proposed to improve the trafficability and driving efficiency of vehicles on rough roads^10^. Sugita proposed a wheel torque distribution algorithm that considers both wheel torque and angular momentum as effectively as possible, which effectively enhances the attitude control angular momentum^11^.

To improve the driving range of EVs, higher requirements are put forward for distributed drive vehicle torque distribution from the perspective of energy. Scholars at home and abroad have done much in this area.

An optimal energy-conserving torque distribution strategy for off-road vehicles with multiple different drivetrains was developed^12^. Dizqah et al. transformed the optimal torque distribution problem into the analytical solution of a parameter optimization problem considering the energy efficiency of the drive chain at different vehicle speeds, which significantly improved the energy efficiency^13^. Optimal torque distribution strategies have been developed to improve the efficiency and endurance mileage of EVs with dual motors^14–17^. A torque distribution control strategy was presented for a four-wheel drive EV to improve the system economy^18^. Torque distribution strategies were reported that ensured the stability and energy savings of EVs with in-wheel motors^19,20^.

The above research focuses on improving the stability and economy of distributed drive vehicles. However, the research objects were ordinary passenger cars. Research on the torque distribution of distributed drive AGVs with variable centroid conditions has rarely been reported. In fact, straight driving or near straight driving is the main part of the driving conditions of an AGV, and the torque distribution strategy under straight driving is crucial.

In this paper, a novel method of AGV torque distribution is proposed to give full play to the advantages of the distributed drive, to maintain AGV straight-line driving and to improve the stability and economy under centroid changes. First, the whole vehicle model of the variable centroid AGV is established, and the driving efficiency characteristics of the hub motor are analyzed. Then, the optimal torque distribution control strategy based on a PIDNN is proposed. Next, a cosimulation platform is built based on CarSim and Simulink to verify the effectiveness of the proposed control strategy. Finally, a model distributed drive AGV is developed to verify the correctness of the simulation results.

The remainder of this paper is organized as follows. The whole vehicle model of the distributed drive AGV is established in “Whole vehicle model under centroid variation” section. The efficiency model of the hub motor is built in “Hub motor efficiency model” section. In “Control strategy” section, the control strategy is proposed. In “Simulation analysis” and “Algorithm verification experiment” sections, simulation analyses and tests of real vehicles are conducted. The conclusions of this paper are given in “Conclusion” section.

## Whole vehicle model under centroid variation

**Figure 1:**
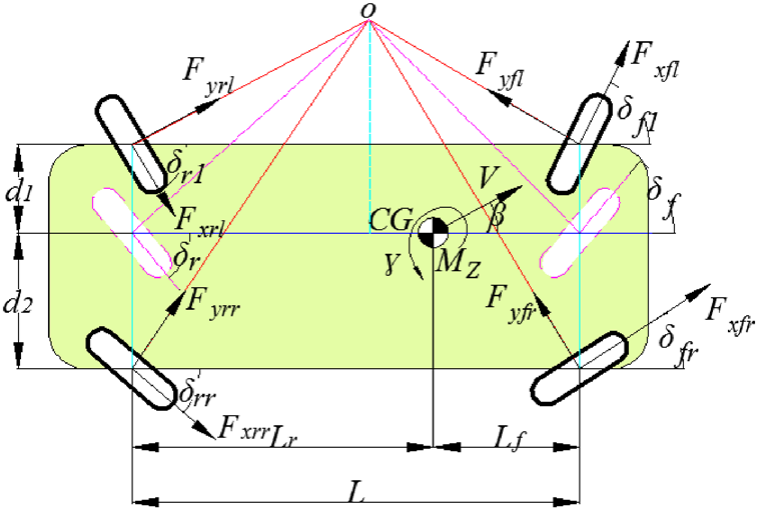
Schematic diagram of the AGV parameters.

Based on the description of the vehicle dynamics process, the vehicle model is simplified to reduce the complexity of the control algorithm. To optimize the torque distribution, three degrees of freedom, including the longitudinal, lateral and yaw movements of the vehicle body, are considered, the vertical, pitch and roll motions of the vehicle are ignored, and the air resistance and other external factors are ignored. Suppose that the AGV runs on a flat road and that the front-wheel and rear-wheel tracks are equal. The AGV dynamic model is established as shown in Fig. 1, where CG is the centroid, *M*_*z*_ is the moment of inertia, and *β* is the sideslip angle of the centroid.

The longitudinal, lateral and yaw motion equations of AGV are as follows:

1 $$ \begin{aligned} m\left( {\dot{v}_{x} - v_{y} \gamma } \right) & = F_{xfl} \cos \delta_{fl} + F_{xfr} \cos \delta_{fr} + F_{xrl} \cos \delta_{rl} + F_{xrr} \cos \delta_{rr} \\ & \quad + F_{yrl} \sin \delta_{rl} + F_{yrr} \sin \delta_{rr} - F_{yfl} \sin \delta_{fl} - F_{yfr} \sin \delta_{fr} \\ m\left( {\dot{v}_{y} + v_{x} \gamma } \right) & = F_{xfl} \sin \delta_{fl} + F_{yfl} \cos \delta_{fl} + F_{xfr} \sin \delta_{fr} + F_{yfr} \cos \delta_{fr} \\ & \quad + F_{yrl} \cos \delta_{rl} + F_{yrr} \cos \delta_{rr} - F_{xrl} \sin \delta_{rl} - F_{xrr} \sin \delta_{rr} \\ M_{z} \dot{\gamma } & = l_{f} \left( {F_{xfl} \sin \delta_{fl} + F_{yfl} \cos \delta_{fl} + F_{xfr} \sin \delta_{fr} + F_{yfr} \sin \delta_{fr} } \right) \\ & \quad + l_{r} \left( {F_{xrl} \sin \delta_{rl} - F_{yrl} \cos \delta_{rl} + F_{xrr} \sin \delta_{rr} + F_{yrr} \cos \delta_{rr} } \right) \\ & \quad + d_{1} \left( {F_{yfl} \sin \delta_{fl} - F_{xfl} \cos \delta_{fl} - F_{yrl} \sin \delta_{rl} - F_{xrl} \cos \delta_{rl} } \right) \\ & \quad + d_{2} \left( {F_{xfr} \cos \delta_{fr} - F_{yfr} \sin \delta_{fr} + F_{xrr} \cos \delta_{rr} + F_{yrr} \sin \delta_{rr} } \right) \\ \end{aligned} $$

where *m* is the mass of the whole vehicle, *v*_*x*_ and *v*_*y*_ are the longitudinal and lateral velocities of the centroid in the vehicle coordinate system, respectively; $\dot{v}_{x}$ and $\dot{v}_{y}$ are the longitudinal and lateral acceleration; *γ* and ${\dot{\upgamma }}$ are the yaw rate and yaw angular acceleration; *F*_*xi*_ and *F*_*yi*_ (*i* = *fl*, *fr*, *rl*, *rr*) are the longitudinal force and lateral force of the tire, respectively; *M*_*z*_ is the moment of inertia of the AGV around the Z-axis; *l*_*f*_ and *l*_*r*_ are the distances from the centroid to the front and rear axles; *d*_*1*_ and *d*_*2*_ are the distance between the equivalent wheel to the left and right wheels, respectively; *δ*_*fl*_ and *δ*_*fr*_ are the left and right steering angles of the front wheels; and *δ*_*rl*_ and *δ*_*rr*_ are the left and right steering angles of the rear wheels.

## Hub motor efficiency model

Four hub motors of the same type are used by the AGV, their efficiency characteristics are basically the same, and their basic parameters are shown in Table 1.

**Table 1:** Hub motor parameters.

Rated voltage/VDC	24–48
Rated output power/W	50–300
Rated torque/(N m), instantaneous maximum torque/(N m)	5, 10
Rated speed/RPM, maximum speed/RPM	400, 600
Tire diameter/mm	200
Mass/kg	3.4

**Figure 2:**
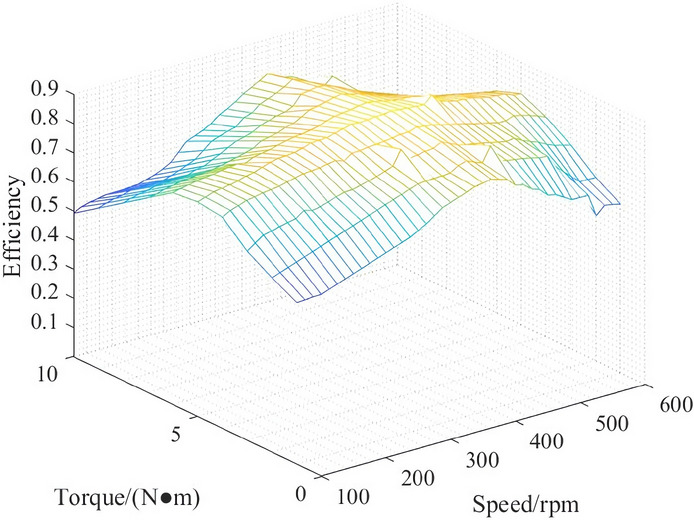
Efficiency characteristics of structure the hub motor.

The motor map is drawn by using the motor data measured under different speeds and torques, as shown in Fig. 2.

Figure 2 shows that the motor efficiency is different under different torques and speeds. Under the same torque, a change in speed has little effect on the motor efficiency, while under the same speed, a change in torque has a more obvious effect on the motor efficiency. According to the distribution of the motor efficiency, when the torque is small or large, the efficiency of the hub motor is low, but the efficiency in the middle area is higher; that is, when the hub motor is under medium load, its efficiency is higher.

Therefore, one of the objectives of the torque control strategy is to reasonably distribute the motor torque so that the hub motor can work in the high-efficiency area as much as possible and improve the vehicle economy.

## Control strategy

**Figure 3:**
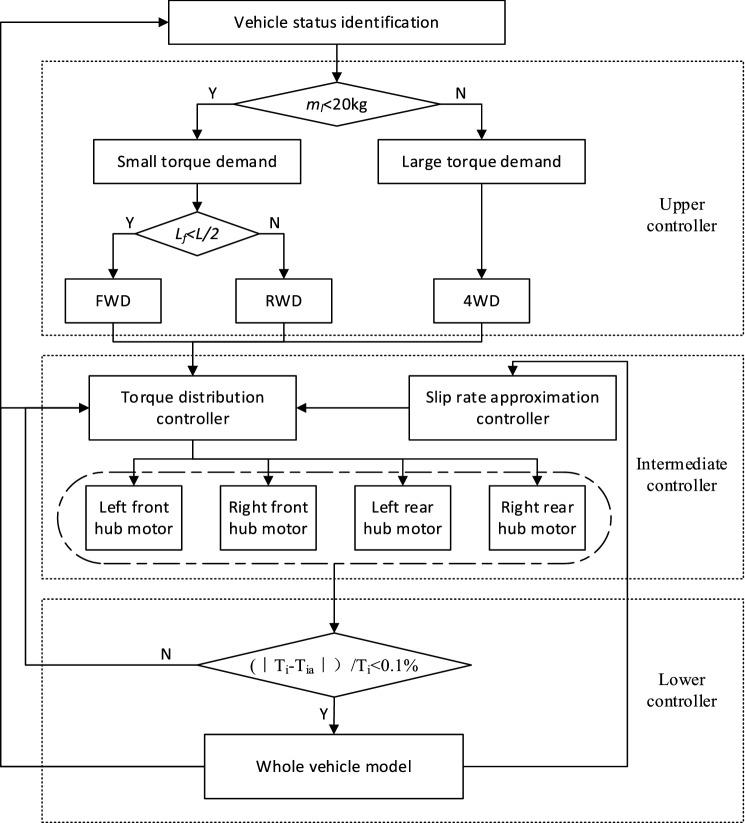
Control strategy diagram.

For the centroid variation straight driving condition, to reduce the energy consumption of the AGV and improve its stability, combined with the characteristics of independent and controllable torque of each driving wheel of distributed driving, a hierarchical control strategy based on a PIDNN is proposed, as shown in Fig. 3.

### Upper controller

In the upper controller, *m*_*l*_ is the load mass, *L*_*f*_ is the distance from the center of mass to the front axle, and *L* is the wheelbase.

The optimal driving mode is determined according to the vehicle parameters and the efficiency model of the hub motor, as shown in Table 2.

**Table 2:** Selection of the best driving mode.

Condition	Optimal driving mode
*m*_*l*_ < 20 kg	RWD/FWD
*m*_*l*_ ≥ 20 kg	4WD
*L*_*f*_ < L/2	FWD
*L*_*f*_ ≥ L/2	RWD

The upper controller determines the required torque of the vehicle according to the AGV payload, and the driving mode is selected. In light of the motor efficiency model, when the hub motor is under medium load, its efficiency is higher.

After several experiments, we notice that when *m*_*l*_ is not less than 20 kg, all hub motors operate with high efficiency under four-wheel drive; when *m*_*l*_ is less than 20 kg, two-wheel drive is most suitable. Therefore, when the load is less than 20 kg, the torque required by the vehicle is small; otherwise, the torque demand is large.

If the demanded torque is small, two-wheel drive is selected to prevent the motors from operating at lower efficiency due to the torque being too small. However, because the position of the centroid of the AGV is uncertain, the choice of front-wheel drive or rear-wheel drive should be considered. Through the experiment, it is found that when the centroid is closer to the front axle, the front-wheel drive mode is more beneficial for the economy of AGVs; otherwise, the rear-wheel drive is selected. If the demanded torque is larger, four-wheel drive is used to prevent the single motor from operating at lower efficiency due to excessive torque.

### Intermediate controller

The intermediate controller combines the torque distribution controller with the slip ratio approximation controller. Within the range of driving forces and under the condition of matching the driving mode determined by the upper controller, the goals are to approximate the slip ratio of the driving wheels, to prevent the wheels from slipping when the vehicle is running, and to improve the vehicle straight-line driving stability. The slip ratio approximation controller works to adjust the torque when the slip ratio difference between wheels is large.

The torque distribution controller calculates the expected torque of each hub motor by the judgment result of the upper controller and the current motion state of the vehicle and then distributes it to each driving wheel.

**Figure 4:**
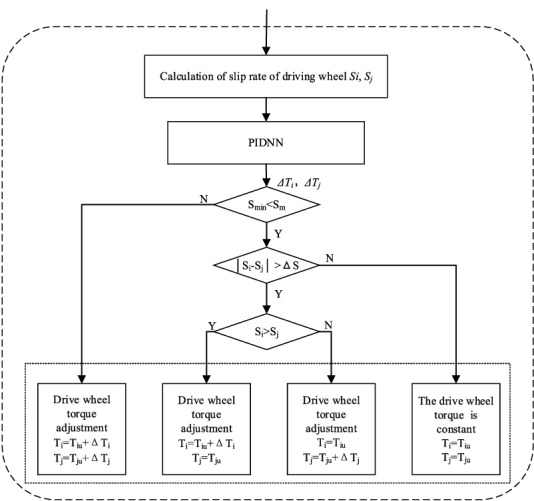
Structure of the slip rate controller.

The slip ratio approximation controller based on the PIDNN calculates the slip ratio of each driving wheel (*S*_*i*_ and *S*_*j*_) by the vehicle speed and wheel speed fed back by the current driving wheel. When the slip ratio approximation controller is working, the torque for antislip control is fed back to the torque distribution controller to adjust the torque of the driving wheels. When it is not working, no feedback is needed. The structure of the slip rate approximation controller is shown in Fig. 4, where *S*_*i*_, *S*_*j*_ and *T*_*i*_, *T*_*j*_ (*i*, *j* = *fl*, *fr*, *rl*, *rr*, *i* ≠ *j*) are the slip ratios and torques of different driving wheels, respectively; *T*_*iu*_ and *T*_*ju*_ are the torques distributed to different drive wheel by the torque distribution controller; and*ΔT*_*i*_ and*ΔT*_*j*_ are the regulating torques of different driving wheels.

Taking the left front wheel of the AGV, its slip ratio is as follows:

2 $$ S_{fl} = \frac{u - r\omega }{u} \times 100{\text{\% }} $$

where *u* is the vehicle speed, *r* is the radius of the driving wheel, and *ω* is the wheel speed, namely, the speed of the hub motor.

The intermediate controller calculates the slip rate of the driving wheels by the vehicle speed and wheel speed, judges whether the slip rates of the driving wheels are too large and whether the slip rates of the driving wheels are similar, then redistributes the torque by the judgment results to ensure the vehicle economy and stability. When the minimum slip rate of the driving wheel *S*_*min*_ is greater than the slip rate threshold *S*_*m*_, the adjusting torque of the driving wheels *ΔT*_*i*_ is output based on the PIDNN algorithm, and the torque of the driving wheels is adjusted at the same time; if the minimum slip rate of the driving wheel *S*_*min*_ is not greater than the slip rate threshold *S*_*m*_ and the difference in the slip rates of the driving wheels is not greater than the set threshold of slip rate difference *ΔS*, the torque of the driving wheels does not need to be adjusted; if the minimum slip rate of the driving wheel *S*_*min*_ is not greater than the slip rate threshold *S*_*m*_ and the slip rate difference of the driving wheels exceeds the set threshold slip rate difference *ΔS*, it is necessary to adjust the torque of the relevant driving wheels.

#### PIDNN algorithm

Research into the torque distribution of distributed drive AGVs under the condition of centroid change must have application value and social significance. The value of the PIDNN lies in its application and practicability; it is necessary for AGV research. The control system designed by the PIDNN has faster real-time performance and stronger adaptability and robustness, and these factors are significant for torque distribution.

**Figure 5:**
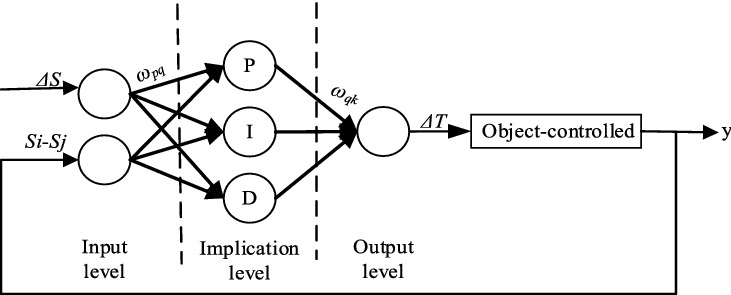
Structure of the PIDNN.

The PIDNN has a three-layer forward network structure, including the input level, implication level and output level; the structure is shown in Fig. 5. The subnetwork is the basic form of the PIDNN control, and the multiple control neural network can be regarded as the combination of several neural network subnetworks. The parallel subnetworks are independent of each other and connected with each other through network weights.

The input layer is the given value and the feedback value of the system, where the given value is the difference threshold of the slip rate of the driving wheels *ΔS* and the feedback value is the difference in the driving wheel slip rates *S*_*i*_-*S*_*j*_. There are three neurons in the hidden layer, namely, the proportional element P, the integral element I and the differential element D. The output layer contains a neuron that is used to receive the calculation results of the hidden layer and add them, ultimately outputting the control law. The PIDNN calculates the error based on the objective function, uses the gradient modification method, constantly modifies the network weights of each layer, calculates the neuron input of each layer, carries out iterative update operations by the neuron update method, and finally obtains the appropriate control rate. In this paper, the output is the adjustable torque of the motor *ΔT*_*i*_. *ω*_*pq*_ is the connection weight between the input layer and hidden layer; *ω*_*qk*_ is the connection weight between the hidden layer and output layer; *p* = 1, 2; *q* = 1, 2, 3; and *y* is the control output.

1. *Input level*
  *X*_*op*_ is the output value of the input layer, *X*_*p*_ is the input value of the system, and *n* is the serial number of the sampled data value:
  3 $$ X_{op} \left( n \right) = X_{p} \left( n \right) $$
2. *Implication level*
  The input value of the implication level is:
  4 $$ N_{q} \left( n \right) = \mathop \sum \limits_{q = 1}^{2} \omega_{pq} X_{op} \left( n \right) $$
  The output values are as follows:
  The proportional element:
  5 $$ N_{o1} \left( n \right) = N_{1} \left( n \right) $$
  The integral element:
  6 $$ N_{o2} \left( n \right) = N_{2} \left( n \right) + N_{2}^{*} $$
  The differential element:
  7 $$ N_{o3} \left( n \right) = N_{3} \left( n \right) - N_{3}^{*} $$
  where $N_{q}^{*}$ is the output value of the neuron calculated in the previous operation step.
3. *Output level*
  The output value is:
  8 $$ U\left( n \right) = \mathop \sum \limits_{q = 1}^{3} \omega_{qk} N_{oq} \left( n \right) $$

### Lower controller

Tracking feedback control is realized by the lower controller. Due to the algorithm, execution structure and other reasons, the torque of the drive wheels deviates from the expected torque. To achieve accurate control, closed-loop tracking feedback control is necessary. The actual control effect of the hub motor is observed and compared with the expected torque. If the relationship between them does not satisfy Eq. (9), the torque of the relevant hub motors must be adjusted to achieve accurate control. If the relationship between them meets Eq. (9), there is no adjustment.

9 $$ \frac{{\left| {T_{i} - T_{ia} } \right|}}{{T_{i} }} < 0.1 $$

where *T*_*i*_ (*i*, *j* = *fl*, *fr*, *rl*, *rr*) is the expected torque value of each driving wheel and *T*_*ia*_ is the actual torque of each driving wheel.

## Simulation analysis

Under the conditions of different loads, CarSim and MATLAB/Simulink are used for cosimulation. Compared with the average torque distribution of the driving wheels, the feasibility of the proposed torque distribution control strategy based on the PIDNN to improve the stability and reduce the energy consumption of the AGV is studied. The parameters of the distributed drive AGV are shown in Table 3.

**Table 3:** Parameters of the distributed drive AGV.

Vehicle curb weight/kg	148
Front and rear wheelbase/m	1.26
Track/m	0.73
Tire radius/m	0.10
Centroid height under no-load conditions/m	0.36
The distance from the centroid to the front axle under no-load conditions/m	0.69
The distance between centroid and rear axle under no-load conditions/m	0.57

### Low load simulation experiment

**Figure 6:**
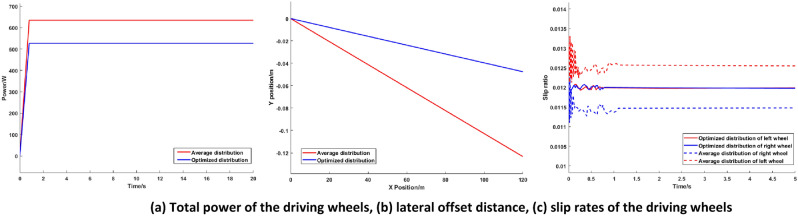
Simulation comparison diagrams of low load conditions.

The speed of the AGV is fixed at 20 km/h, the load weight is 10.2 kg, and the load is placed in the right front corner of the load platform. The vehicle runs on a straight level road. The simulation of the torque optimization distribution of the AGV based on the PIDNN is carried out, and the simulation results are compared with the average torque distribution effect (in this paper, all “average torque distributions” are equal distributions to all driving wheels). The simulation results are shown in Fig. 6.

The simulation results show that under low load conditions, the torque demand of the whole vehicle is lower, and the centroid of the whole vehicle is closer to the front axle. To increase the possibility of the motor working at high efficiency, FWD is used for the AGV. It can be seen from Fig. 6a that the total power of the driving wheels of the optimized distribution (in this paper, all “optimized distributions” are the torque optimized distributions based on the PIDNN) is 17.63% lower than that of the average torque distribution, which indicates that under low load conditions, the proposed optimized distribution can achieve the optimal motor utilization efficiency, reduce energy consumption and improve the economy of the AGV. Figure 6b shows that when the X-axis position of the AGV is 125 m, the Y-axis position of the AGV is reduced from 0.13 m with the average torque distribution to 0.05 m with the proposed optimized distribution, and the lateral deviation distance is significantly reduced. As shown in Fig. 6c, in the first second, the slip rate of the driving wheels fluctuates greatly. After one second, due to the effect of the control strategy, the slip rate tends to be stable. In the first second, compared with the average torque distribution, the slip rate of the optimized distribution is lower and more stable, and the slip rate of the optimized distribution tends stabilize more quickly. The slip rates of the left and right driving wheels of the distributed drive AGV with equal torque distribution to all driving wheels are not equal, and the difference is large. At this point, the AGV is under extremely unstable conditions. The torque distribution control strategy based on the PIDNN proposed in this paper reduces the slip rate of the left driving wheel and increases the slip rate of the right driving wheel to achieve the goal of similar slip rates for the driving wheels and improve the stability on the basis of an AGV driving on a straight path.

The simulation results under low load conditions show that the proposed torque optimal distribution control strategy based on the PIDNN can effectively reduce energy consumption, reduce the lateral deviation distance of the AGV and improve the stability of the vehicle compared with those of the average torque distribution control.

### High load simulation experiment

**Figure 7:**
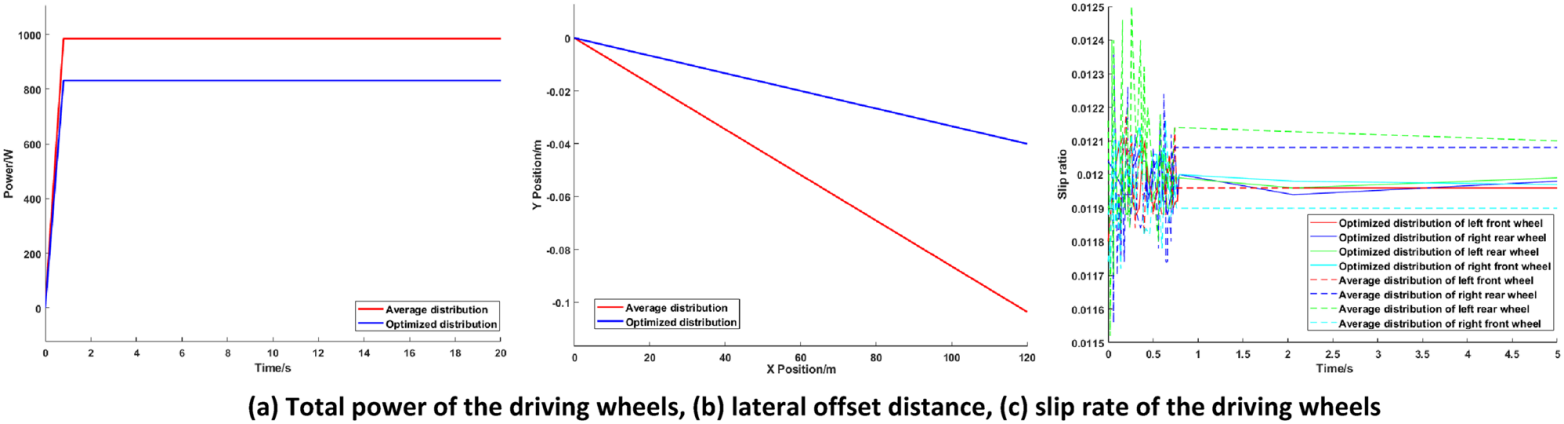
Simulation comparison diagrams of high load conditions.

The speed of the vehicle is fixed at 20 km/h, the load weight is 27.2 kg, and the load is located on the left rear of the platform. The vehicle runs on a straight level road. Simulation of the torque optimization distribution of the AGV based on the PIDNN is conducted, and the simulation results are compared with the average torque distribution. The simulation results are shown in Fig. 7.

The simulation results show that under high load conditions, the vehicle torque demand is large. To prevent excessive torque in a single motor, reducing the motor efficiency, four-wheel drive is used for the AGV. Compared with that of equal torque distribution to all driving wheels, the total power of the driving wheels of the optimized distribution is 15.54% lower, which indicates that the optimal torque distribution based on the PIDNN can achieve the optimal utilization efficiency of the motor under high load conditions. It can be seen from Fig. 7 that in the two distribution methods, the optimal torque distribution control based on the PIDNN reduces the lateral deviation distance of the AGV by 61.39%, the slip rates remain close, and the vehicle stability is better.

The results of high load simulation show that the proposed torque optimal distribution control based on the PIDNN can effectively reduce the energy consumption and the lateral deviation distance of the AGV, keep the slip ratio of the driving wheels close, and improve the economy and stability of the AGV.

Through the joint simulation of CarSim and MATLAB/Simulink, it can be found that under different load conditions, the total power of the driving wheel and lateral offset distance of the AGV are reduced to varying degrees, and close slip rates are realized, which proves that the proposed control strategy can effectively improve the economy and stability of the AGV under different mass center conditions. Moreover, in the low load and high load experiments, the loads are placed in challenging positions, and the vehicle can still maintain a stable straight-line driving state with good economy. Therefore, the effectiveness of the proposed control strategy is further confirmed.

## Algorithm verification experiment

### Sample machine development

**Figure 8:**
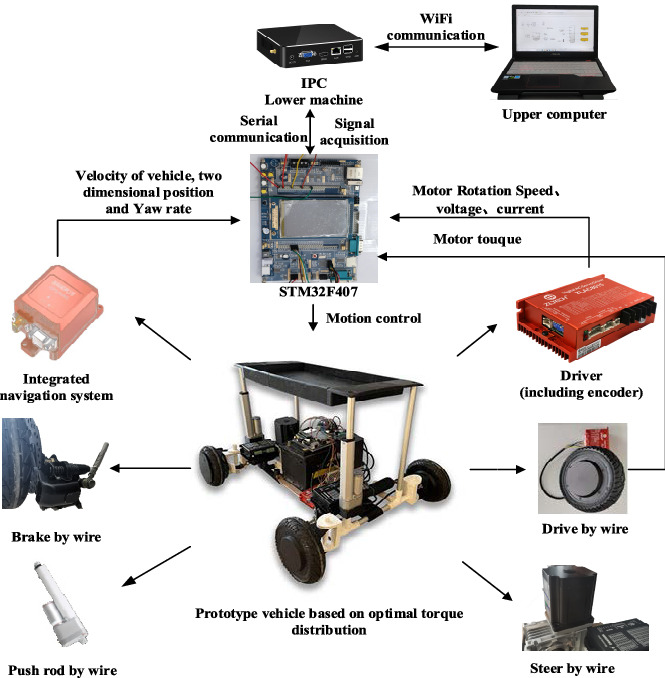
Experimental platform.

To verify the effectiveness of the proposed optimal torque distribution control strategy based on the PIDNN and the correctness of the simulation results, a model distributed drive AGV is developed, as shown in Fig. 8.

The prototype carries an STM32F407 for the lower computer and an embedded mini industrial control computer for the upper computer. Each motor is manipulated by controlling the motor driver of the driving, steering and braking system of the AGV to realize the motion control of the AGV. The position and pose information of the vehicle, such as the two-dimensional position, vehicle speed and yaw rate, is determined by a high-precision INS inertial navigation system. The real-time parameters of the wheel motors, such as the motor speed and torque, are collected by the motor encoder and torque sensor, respectively. The real-time signal is collected by the STM32F407 and fed back to the upper computer. The upper computer sends out a control command to the single-chip microcomputer through serial communication, and the single-chip microcomputer directly controls the corresponding motors according to the command.

### Algorithm verification experiment under centroid variation

**Figure 9:**
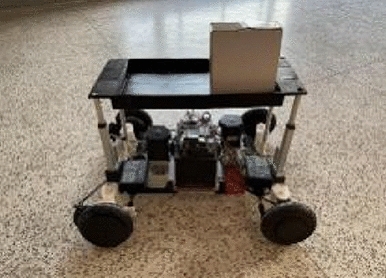
Low load test.

**Figure 10:**
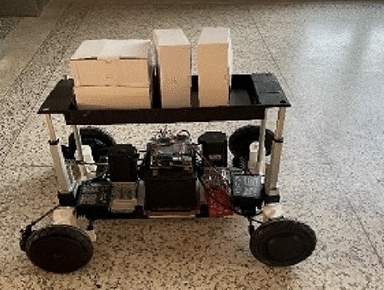
High load test.

To simulate the transport environment of intelligent factories, algorithm verification experiments are conducted under different load conditions. The vehicle speed is 20 km/h, and the parameters and load position of the AGV are consistent with those used in the simulation. The experimental figures are shown in Figs. 9 and 10.

During the experiment, to reduce the error, several groups of experiments are carried out, and the average value is calculated to verify the effectiveness of the proposed control strategy. By multiplying and summing the collected motor bus current and bus voltage, we can obtain the sum of the instantaneous power of the working motor, namely, the total power of the driving wheels, as shown in (a). The two-dimensional position of the AGV can be measured through the inertial navigation system, as shown in (b). The vehicle speed can be measured through the inertial navigation system, and the speed of hub motor can be measured through the driver of hub motor, then the slip rate of the driving wheels can be obtained, as shown in (c).

**Figure 11:**
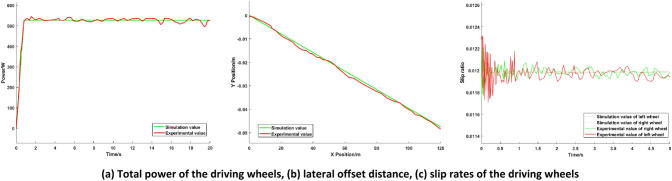
Experimental comparison diagrams of low load conditions.

**Figure 12:**
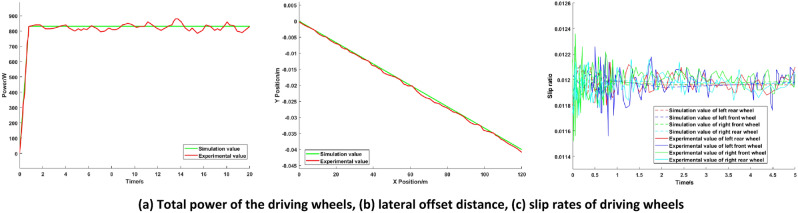
Experimental comparison diagrams of high load conditions.

The experimental results are shown in Figs. 11 and 12.

Figures 11 and 12 show the total power, lateral offset distance and slip rates of the driving wheels under different load conditions. It can be seen from Figs. 11 and 12 that the experimental results are basically consistent with the simulation results, and the deviation is kept within 5%, which shows that the simulation analysis of the control strategy is correct and further confirms the effectiveness and correctness of the proposed torque optimal distribution control strategy based on the PIDNN.

## Conclusion

1. Under the centroid variation premise, taking the distributed drive AGV as the research object, the whole vehicle dynamics model is established, which provides a theoretical basis for establishing the model in Simulink.
2. This paper analyzes the relationship between the drive efficiency of the hub motor and the motor speed and torque, establishes an efficiency model of the hub motor, provides ideas for energy-saving torque distribution, and analyzes the feasibility of achieving the lowest energy consumption through reasonable torque distribution.
3. To improve the economy and stability of AGVs under straight-line driving conditions, an optimal torque distribution control strategy based on a PIDNN is proposed. The control system includes an upper controller, intermediate controller and lower controller. The driving mode is determined in the upper controller, the torque of driving wheels is adjusted in the middle controller to achieve the goal of similar slip rates, and tracking feedback control is realized in the lower controller to ensure accurate control.
4. Based on CarSim and MATLAB/Simulink, a cosimulation platform is established for testing. The vehicle model and simulation conditions are established in CarSim, and the motor and control system models are established in Simulink. The cosimulation results show that compared with that of the average torque distribution, the total power of the driving wheels is reduced by 17.63% and 15.54% under low load and high load conditions, respectively. With the proposed torque optimization and distribution control strategy based on the PIDNN, the lateral deviation distance is reduced by 61.47% and 63.39%, and the slip rates of the driving wheels can be kept close. It is proven that the proposed control strategy is effective.
5. Algorithm verification experiments are conducted, and a prototype of a distributed drive AGV is developed to simulate an intelligent factory transport environment. Algorithm verification experiments are conducted under different load conditions. The experimental results are basically consistent with the simulation results, and the deviation is less than 5.8%, which verifies the correctness of the algorithm.

## Data Availability

The datasets supporting the conclusion of this article are included within the article.
